# Kinematics and energetics of foraging behavior in Rice’s whales of the Gulf of Mexico

**DOI:** 10.1038/s41598-023-35049-z

**Published:** 2023-06-02

**Authors:** Annebelle C. M. Kok, Maya J. Hildebrand, Maria MacArdle, Anthony Martinez, Lance P. Garrison, Melissa S. Soldevilla, John A. Hildebrand

**Affiliations:** 1 Scripps Institution of Oceanography, University of California San Diego, La Jolla, CA 92093 USA; 2 Marine Mammal and Turtle Division, Southeast Fisheries Science Center, National Marine Fisheries Service, Miami, FL USA

**Keywords:** Behavioural ecology, Conservation biology

## Abstract

Rorqual foraging behavior varies with species, prey type and foraging conditions, and can be a determining factor for their fitness. Little is known about the foraging ecology of Rice’s whales (*Balaenoptera ricei*), an endangered species with a population of fewer than 100 individuals. Suction cup tags were attached to two Rice’s whales to collect information on their diving kinematics and foraging behavior. The tagged whales primarily exhibited lunge-feeding near the sea bottom and to a lesser extent in the water-column and at the sea surface. During 6–10 min foraging dives, the whales typically circled their prey before executing one or two feeding lunges. Longer duration dives and dives with more feeding-lunges were followed by an increase in their breathing rate. The median lunge rate of one lunge per dive of both animals was much lower than expected based on comparative research on other lunge-feeding baleen whales, and may be associated with foraging on fish instead of krill or may be an indication of different foraging conditions. Both animals spent extended periods of the night near the sea surface, increasing the risk for ship strike. Furthermore, their circling before lunging may increase the risk for entanglement in bottom-longline fishing gear. Overall, these data show that Rice’s whale foraging behavior differs from other lunge feeding rorqual species and may be a significant factor in shaping our understanding of their foraging ecology. Efforts to mitigate threats to Rice’s whales will benefit from improved understanding of patterns in their habitat use and fine-scale ecology.

## Introduction

The Rice’s whale (*Balaenoptera ricei*) is the only baleen whale species that is resident in the northern Gulf of Mexico (GOM)^1^. Genetic and morphological evidence recently indicated that the Rice’s whale is a unique species, separate from the Bryde’s whale complex^1,2^. Due to their small population size^3^ and exposure to anthropogenic threats, they were listed as an endangered species in 2019. However, devising conservation measures remains difficult, in part because little is known about Rice’s whale foraging ecology. The availability of prey and the effort required to obtain that prey can directly affect an animal’s fitness, and these factors are a known cause of decline for other endangered species, such as killer whales^4^. Rice’s whales, like other balaenopterid whales or rorquals, forage by lunge-feeding, primarily while breath-holding at depth^5,6^. Lunge-feeding involves engulfing a large volume of water and prey, while stretching the buccal cavity along a series of longitudinal throat grooves^5^. The water is then expelled, trapping the prey against the fringed baleen. Rice’s whales are thought to feed on pelagic schooling fishes^7^ but the details of their prey, foraging behaviors and energetics remain unclear. To unravel one of the possible causes for the low population number of Rice’s whales, we investigated the kinematics and energetics of their foraging behavior.

Lunge feeding is a widespread foraging method among rorquals. Because of the large volume of prey that can be consumed in one gulp, it is very energetically efficient, and has likely been one of the drivers of gigantism in baleen whales^8^. Baleen whale species show positive allometry of the buccal cavity, which leads to a higher mass-specific energy acquisition for larger species^9^. This higher acquisition per lunge comes at a cost. Large whale species have higher mass-specific energetic costs per lunge, limiting the number of lunges they can make per dive^9,10^. Another factor influencing the number of lunges per dive is prey depth, with deeper diving whales increasing the number of lunges per dive to compensate for increased transit cost^11–13^. Smaller rorquals, such as Rice’s whales, are expected to be able to make more lunges per dive than their larger counterparts, assuming they dive to similar depths^5^. However, most of those theories were derived from rorquals foraging on krill, and might be different for fish eating rorquals.

The main prey items of rorquals are zooplankton and small schooling fish. While zooplankton does not escape the attacks of their predator, schooling fish do, increasing the energy expenditure and changing the foraging tactics required by the rorquals that target them. Rorquals foraging on fish may spend time chasing their prey before lunging^14^, and can reach higher speeds during the lunge than rorquals feeding on krill^15–17^. This higher energetic cost is compensated for by the higher caloric content of fish prey, but the compensation depends on prey availability and the energy required to switch between patches^18^. Even though the targeted prey type can change over time^18^, an overall decrease in prey availability could thus lead to lower energy gains.

Multiple rorqual species were seen to vary their foraging tactic with available prey type and behavior^15,16,19–22^. Blue, fin, and humpback whales show multiple orientations of lunge feeding—vertical, lateral and inverted^23–25^. Humpback whales often herd their prey before lunging^24,26–28^, and for some fish prey, they abandon lunging in favor of bottom side roll feeding^29^. Bryde’s whales, a sister species of Rice’s whales, show a variety of foraging tactics that differ with prey type and population. Bryde’s whales in New Zealand adjusted the orientation of their surface lunges depending on the prey type (krill vs fish)^15^, while Bryde’s whales in the western Pacific did not lunge but foraged by tread-water feeding^30,31^. A third type of foraging was observed in Bryde’s whales off the coast of South Africa, where animals performed high-speed chases close to the seafloor before engulfing their fish prey^14^. Foraging strategies of Rice’s whales are likely to be similarly influenced by the available prey type and behavior in their core area.

The aim of this study was to investigate how foraging kinematics of the critically endangered Rice’s whale fit in the current theoretical framework of rorqual foraging. Specifically, we investigated (1) the number of lunges per dive, (2) the energy expended during each part of a foraging dive, and (3) how energy expenditure related to breathing rate. This study presents the first detailed description of the Rice’s whale foraging kinematics and energetics and provides insight into the current and future challenges to the survival of the species.

## Results

### General diving behavior

Two Rice’s whales were tagged with suction-cup Acousonde tags (Greeneridge Sciences, Inc.) that supported a hydrophone, pressure sensor, accelerometer and magnetometer. One animal was tagged in 2015, known in the photo ID record as ‘Milky Way’ (Catalog ID 20014), and one animal was tagged in 2018, known as ‘Edna’ (Catalog ID 12003). The tag attached to Milky Way remarkably stayed on the whale for ~ 64 h, providing nearly three complete daily behavioral cycles. The tag attached to Edna also remained on the whale for an extended period of ~ 25 h (Table 1). Milky Way was first seen at the time of the tag attachment and was resighted again in summer of 2019. Milky Way was paired with a smaller (perhaps juvenile) whale at the time of tag attachment, and presumably is a female. A more detailed account of the tagging procedure and a dive profile have been previously reported for this animal^6^. Edna had first been sighted earlier in 2018 and was resighted again in 2019. The whale is of unknown sex. The body conditions of both whales appeared to be fair to lean; they were thin with prominent dorsal vertebrae (Ruth Ewing pers. comm.).

**Table 1:** Diving, breathing and lunging rates for tagged Rice's whales.

	Tag on (h)	Tag off (h)	Total lunges	Total Dives > 10 m	Dives > 10 m/h (day)	Dives > 10 m/h (night)	Breaths/h (day)	Breaths/h (night)	Lunges/h (day)	Lunges/h (night)
'Milky Way'	20 Sept 2015 09:25 CDT (0)	23 Sept 2015 01:10 CDT (63.8)	133	217	4 (1–7)	2 (0–11)	35 (2–65)	26.5 (5–43)	4 (0–8)	0 (0–10)
'Edna'	3 July 2018 17:16 CDT (0)	4 July 2018 18:25 CDT (24.9)	135	137	7 (5–11)	2 (0–7)	83 (12–98)	66 (48–81)	9 (2–11)	0 (0–6)

The hour of sunset was included in the night hours, while the hour of sunrise was included in daylight hours. Incomplete hours due to tag on or tag off times were discarded when calculating hourly rates. For hourly rates, values shown are median (range).

**Figure 1:**
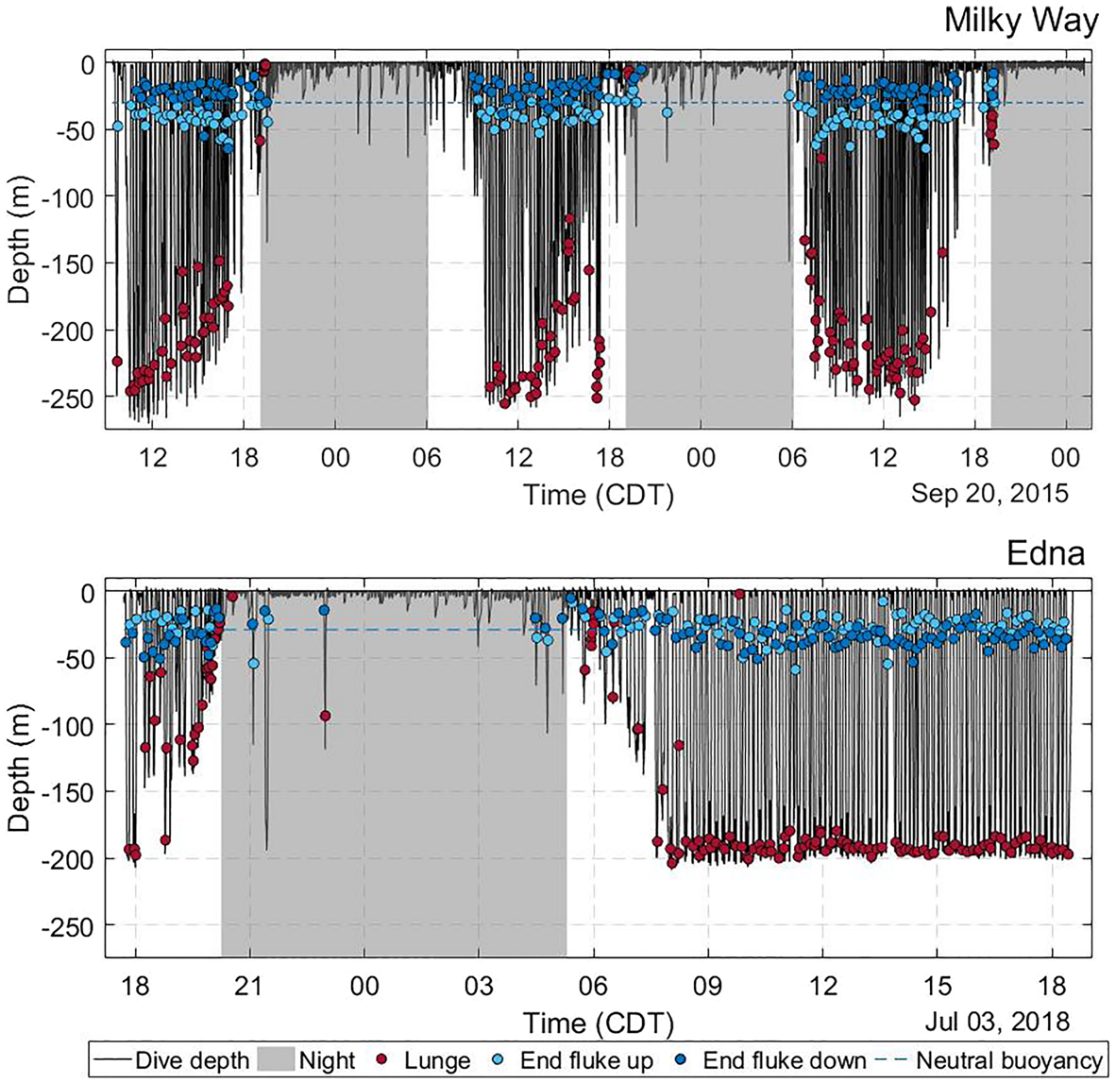
Dive patterns (black line) of Rice's whales (top) Milky Way and (bottom) Edna. Period between sunset and sunrise shaded gray. Dots depict foraging lunges (red), depth of fluking cessation during ascent (light blue), and descent (dark blue). Blue dashed line designates median neutral buoyancy based on the cessation of fluking during descent and ascent. Note how Milky Way ceased fluking at a shallower depth on the descent vs the ascent (dark blue dots above dashed line, light blue dots below dashed line), while Edna fluked to the same depth on both descent and ascent (light blue and dark blue dots typically at same depth).

Both tagged whales had diel dive patterns with deep dives (> 100 m) throughout the day that became shallower during twilight and remained near the surface throughout most of the night (Fig. 1). The seafloor in the general area around the tagging location of Milky Way was 230–270 m deep (256 m at the tagging location) and was 200–230 m deep around the location of Edna (202 m at the tagging location). The tagged animals both dove to depths near the seafloor, and foraging lunges were identified from the accelerometer and speed measurements at the deepest parts of their dives. The lack of variability in maximum dive depths of Edna (all ~ 200 m) suggested that Edna tended to closely adhere to the sea bottom during the day (Fig. 1). Most foraging dives occurred during daylight hours. At night, the whales stayed close to the sea surface, typically making shallow dives (< 10 m) with occasional dives to moderate depths (~ 100 m) but with few or no foraging lunges. A trend for increasing foraging dive depth during the morning and decreasing foraging dive depth at night suggests that these whales were likely foraging on diel vertically migrating organisms.

### Lunging behavior

Dive cycles exhibited similar patterns for both whales. Foraging dives followed stereotyped patterns and are defined here as any dive to a depth of at least 10 m that contained a foraging lunge (Fig. S1). The average reported length for a Rice’s whale is 9.2 m, so with a dive depth of 10 m or more the whale was at least one body length away from the surface. For each dive, after a burst of fluking near the surface, the whale reached the depth of neutral buoyancy and then glided to the depth at which it began foraging.

**Figure 2:**
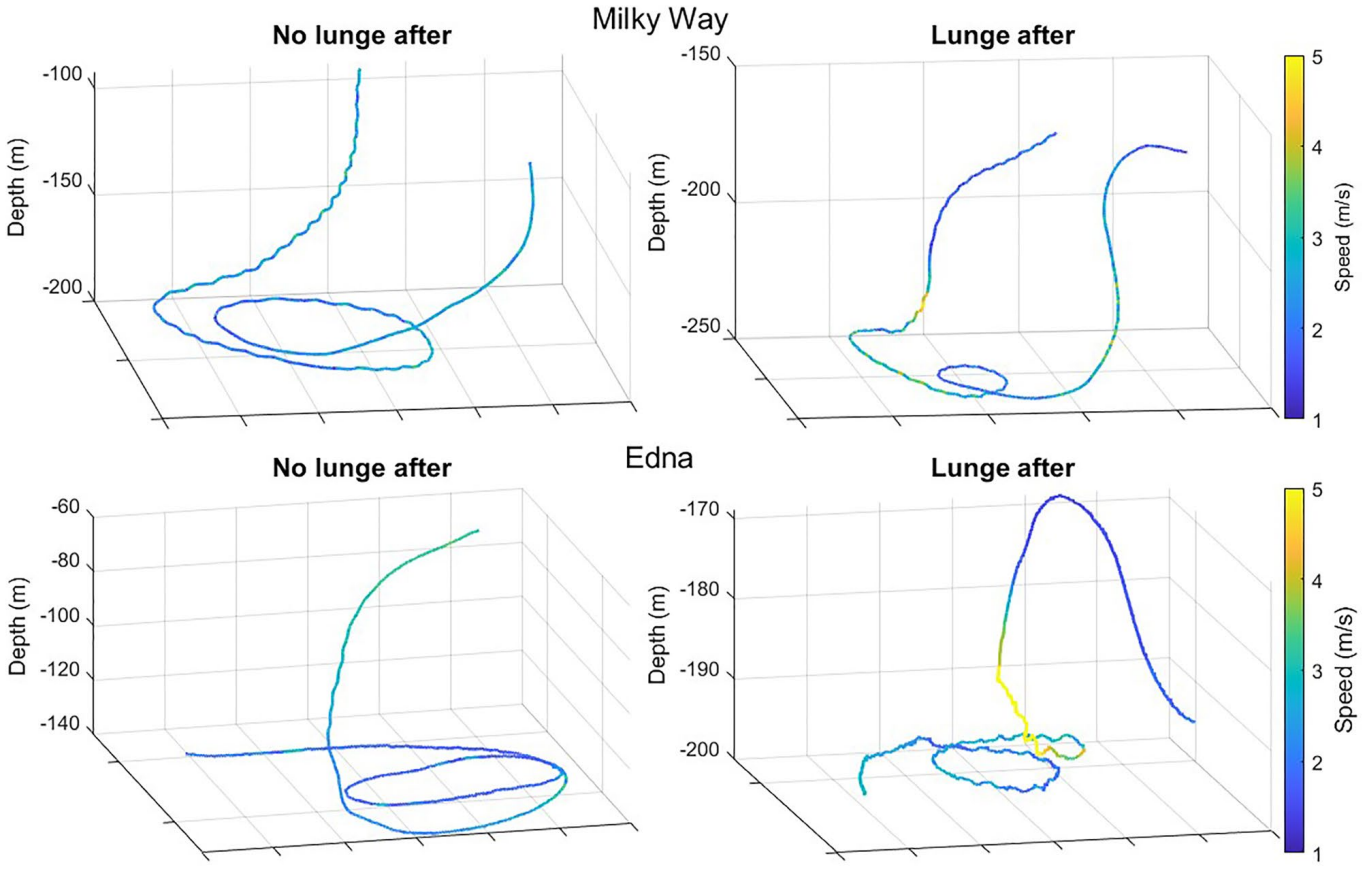
Circling behavior of Milky Way (top) and Edna (bottom). Some circling was not followed by lunging (left) while other circling was (right). Note how the animals were staying level while circling and only increased speed during circling when the circling was followed by a lunge.

Prior to a lunge, both animals performed circling behavior identified by a continuous ≥ 180° rotation in heading, while pitch and roll remained constant (Fig. 2, Fig. S3). Milky Way started with circling before a lunge in 47% of lunges, while Edna started with circling before a lunge in 77% of lunges. Milky Way circled for 23–446 s, while Edna circled for 3–21 s. For both animals, lunges preceded by circling were at greater depth than lunges without circling (median lunge depth with vs without circling, Milky Way = 197 vs 179 m, Edna = 154 vs 44 m; linear model (LM), circle: estimate = 109.1, p < 0.0001). Both animals sometimes circled without lunging. In about 20% of circling events for Milky Way and 9% of circling events for Edna, circling was not followed by a lunge, giving a ratio of presumably failed foraging attempts. When a lunge followed, both animals were more likely to speed up toward the end of circling, compared to when they did not lunge.

**Figure 3:**
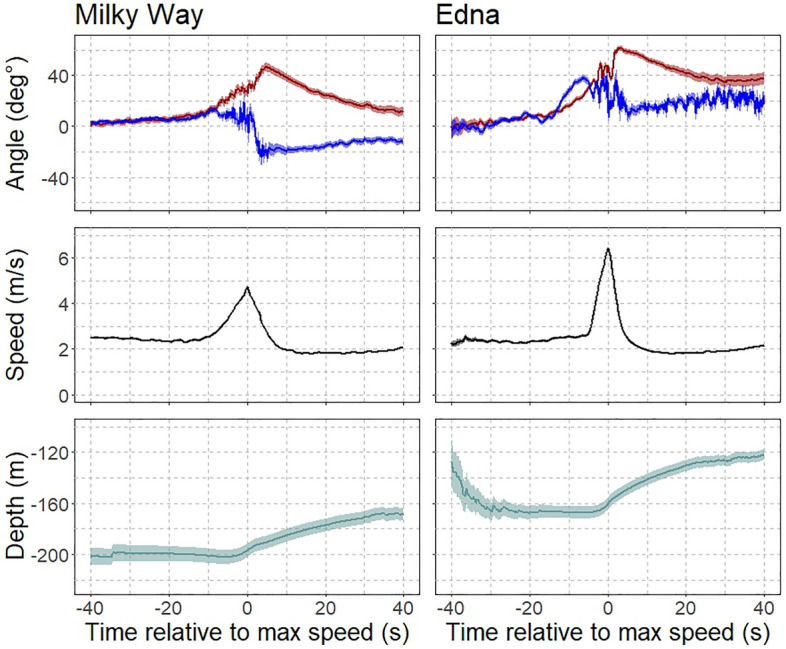
Average foraging lunges of Milky Way (left) and Edna (right). Both animals typically pitched upward (top panel, red line) until after the maximum speed was reached (middle panel) and then proceeded to angle down. During the last part of the upward pitch, Milky Way started rolling (top panel, blue line), a feature that was more variable and earlier in the lunge for Edna. During a lunge, the speed (middle panel, black line) showed a sharp increase and decrease for both animals. Both animals typically foraged at depth (bottom panel, green line), going up in the water column just before reaching maximum speed. Lines represent mean values, shaded areas around the lines show 5–95% confidence intervals.

Lunging behavior followed a stereotypical pattern for both whales. A typical lunge started with an increase in pitch (changing orientation to point upward) and speed, with a strong (~ 30°) roll of their body starting just before the speed peaked (Fig. 3). Lunges were predominantly conducted at depths > 50 m (90% of lunges). The peak in speed coincided with an increase in pitch and a decrease in depth, indicating that the whale was lunging upward in the water column. After the maximum speed was reached, it quickly dropped down below pre-lunge levels. The pitch, meanwhile, increased until around halfway through the speed decrease, at which point it switched direction, indicating that the whale was angling down. These patterns were similar between the two whales, although Edna showed more variability in the roll and tended to roll to the right initially, while Milky Way rolled to the left.

The number of foraging lunges per dive occurred with a median (range) of 1 (1–3) lunge per dive for Milky Way and 1 (1–6) lunges per foraging dive for Edna (Table 2). While both animals averaged one to two lunges per foraging dive (night foraging included, Wilcoxon, W = 4392, p = 0.8, Table 2), Edna’s daylight dives were shorter (379 vs 522 s, Table 2) and had a higher percentage of foraging compared to non-foraging dives (percentage of daylight dives with foraging: Edna: 80%, Milky Way: 57%; Wilcoxon, W = 10,594, p < 0.0001). This led to Edna performing nearly four times as many foraging lunges per hour as Milky Way (8.1 vs 2.4; Table 1). On average, Milky Way had deeper foraging dives (206.8 m vs 172.5 m; Wilcoxon, W = 4033.5, p < 0.0001) and was submerged twice as long during each dive compared to Edna (Wilcoxon, W = 4490.5, p < 0.0001, Table 2).

**Table 2:** Foraging dive characteristics for Rice's whale dives with depth > 10 m, mean ± se.

	Dives with lunge	Dive depth (m)	Submerged time (s)	Descent rate (m/s)	Descent speed (m/s)	Ascent rate (m/s)	Ascent speed (m/s)	# Breaths/dive	Breathing rate	# Lunges/dive
Milky Way	92	206.8 ± 6.6	521.7 ± 21.0	0.9 ± 0.004	2.0 ± 0.003	1.6 ± 0.005	2.2 ± 0.002	8.6 ± 0.5	2.3 ± 0.1	1.3 ± 0.06
'Edna'	96	172.5 ± 4.6	378.9 ± 10.2	2.0 ± 0.01	2.7 ± 0.004	1.9 ± 0.01	2.5 ± 0.004	11.8 ± 1.3	5.8 ± 0.5	1.4 ± 0.05

Note that descent/ascent rate is change in depth over time, while speed is the average speed during descent/ascent.

**Figure 4:**
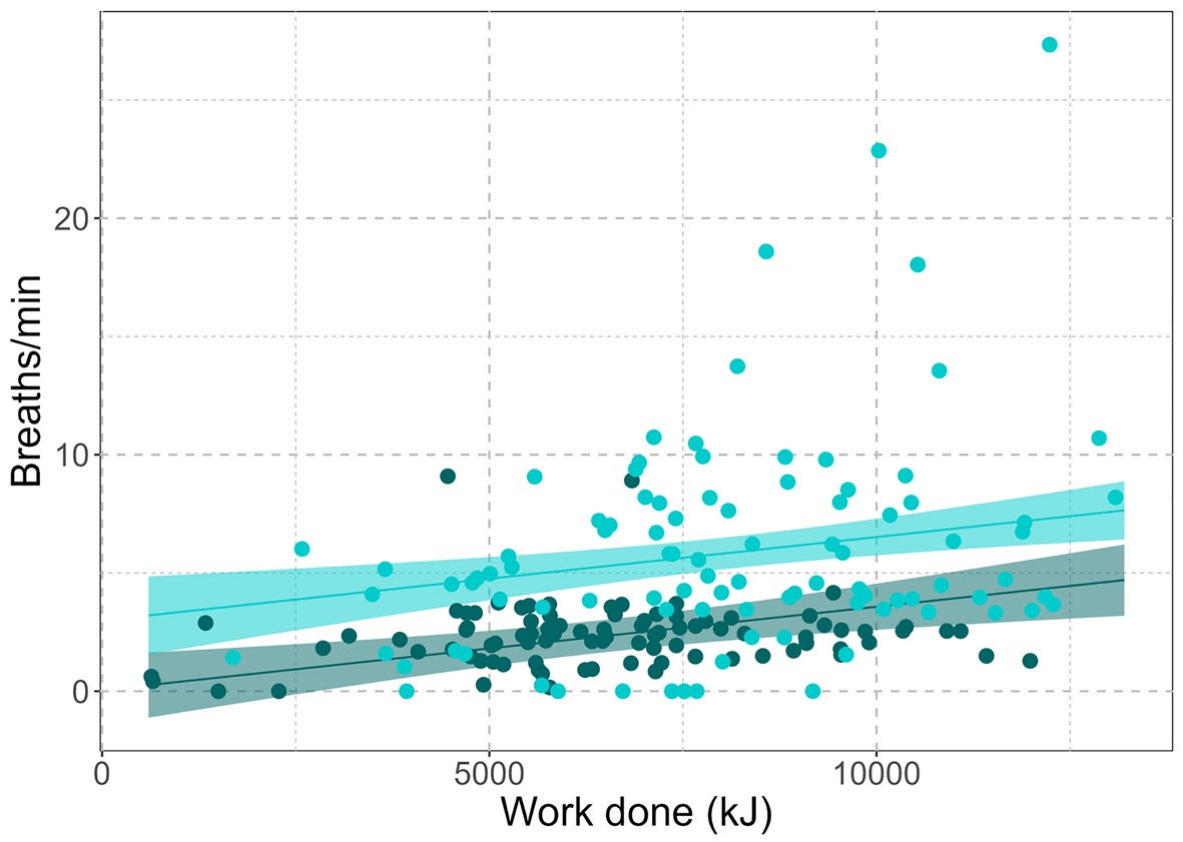
Both animals had a higher breathing rate after spending more energy during a foraging dive. ‘Edna’ (turquoise) had a breathing rate that was on average twice as high as the breathing rate of ‘Milky Way’ (dark green). Dots represent single dive and surfacing events, lines show generalized linear smooths per individual with a 95% confidence interval (shaded area).

At the end of the dive, the whales ascended by fluking to reach the point of neutral buoyancy, after which they glided to the surface. The whales took a series of breaths at the surface which appeared as impulsive sounds in the acoustic recordings (Fig. S1). While Milky Way made longer and deeper dives, Edna had a breathing rate that was significantly higher than that of Milky Way (5.8 breaths/min vs 2.3 breaths/min, Table 2, Fig. 4) and took more breaths during each surfacing event (11.8 vs 8.6, Table 2). Furthermore, Edna had higher descent and ascent rates compared to Milky Way (1.1 m/s higher on the descent and 0.3 m/s higher on the ascent, Table 2).

### Diel patterns in foraging

**Figure 5:**
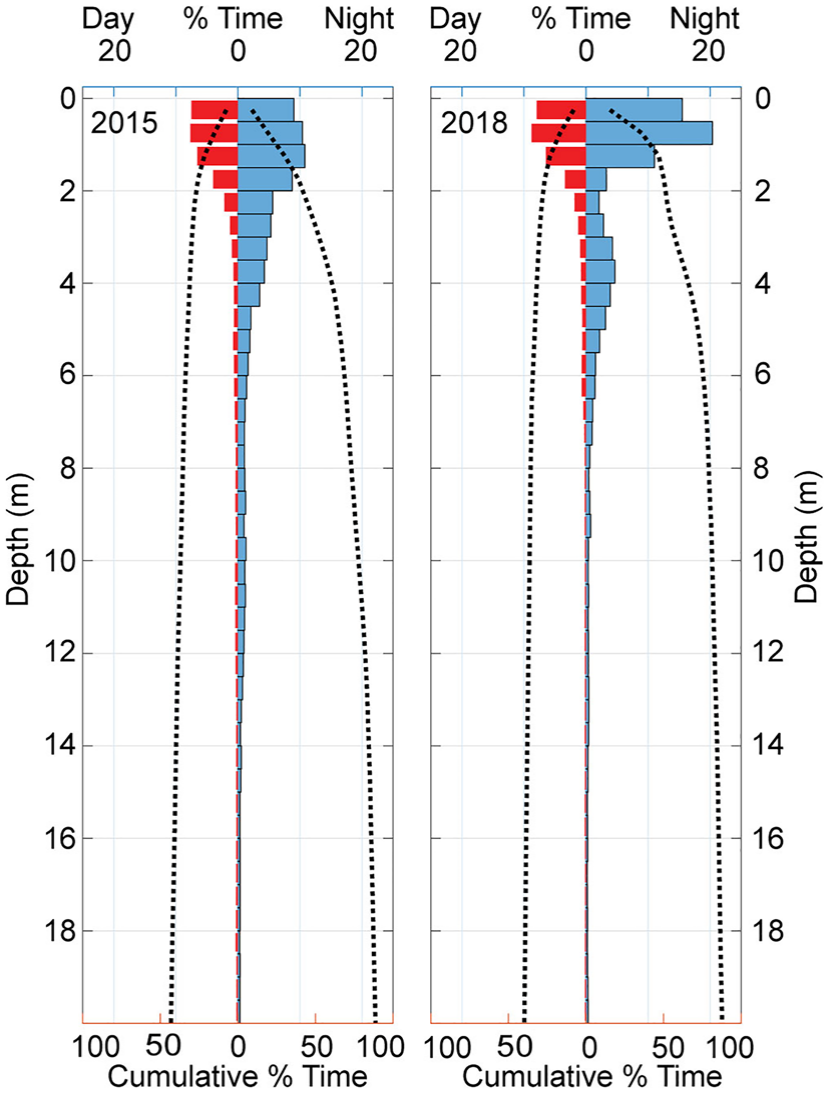
Percent of time spent at depths 0–20 m during daylight (red) and nighttime (blue) for Milky Way (2015) and Edna (2018), along with cumulative time spent at a given depth or shallower (dotted lines).

Both animals showed consistent foraging activity during daylight involving deep dives with typically one lunge per dive (Fig. 1). During the nighttime, both whales remained near the sea-surface, except for an occasional dive to 50–100 m depth (Fig. 5). Both animals only used a small percentage of the dives at night for foraging (Edna: 2/21 = 9.5%, Milky Way: 7/76 = 9.2%; Wilcoxon, W = 800.5, p = 0.97). Milky Way made seven surface foraging lunges and eleven deep foraging lunges at night, while Edna made one surface and six deep foraging lunges at night. All surface foraging lunges were vertical, similar to the deep foraging lunges. Neither whale made any surface lunges during daylight. During nighttime, both whales were within 15 m of the sea surface 85% of the time and within 2 m of the surface 45% (Milky Way) and 52% (Edna) of the time (Fig. 5).

### Neutral buoyancy

The point of neutral buoyancy is the depth at which the body density of a marine animal is equal to the surrounding water. Besides the air in the lungs, an important factor in body density of marine mammals is the size of lipid stores, which are less dense than the other tissues and water and therefore reduce body density. Individuals with large lipid stores will be more positively buoyant, while their leaner counterparts will be more negatively buoyant. Their buoyancy influences their locomotion patterns. Gliding is most efficient when the net buoyancy aids movement, i.e. a negatively buoyant animal can easily glide downward, while a more positively buoyant animal has to fluke to get to deeper depths^32,33^. Differences in buoyancy between animals can thereby drive differences in fluking and gliding patterns during dives^32–36^.

Both animals glided during the majority of the descent. Using the average of the descent and ascent depths at which each animal ceased fluking as an estimate for their depth of neutral buoyancy, Edna reached neutral buoyancy at ~ 26 m and Milky Way reached neutral buoyancy at ~ 31 m (Wilcoxon, W = 19,946, p < 0.001; Fig. 1). However, Milky Way consistently stopped fluking at a shallower depth on the descent than on the ascent, whereas Edna continued fluking to a similar depth on both ascent and descent (Fig. 1). The additional fluking resulted in Edna having a higher descent and ascent rate and spending less time in the descent and ascent phases (Table 2).

### Energy expenditure

Foraging efficiency is determined from the ratio between energy intake and energy expenditure. If the energy intake is smaller than the energy expenditure, the ratio is < 1, so energy is being depleted. If the intake is greater than the expenditure, the ratio is > 1, and energy is being stored. If the ratio is exactly 1, the foraging animal is only able to obtain enough food to sustain itself, but it is not able to store energy for future needs, including breeding. The energy expenditure during foraging consists of two components: the basal metabolic rate (BMR) of the animal, and the work done, i.e. the extra energy expended because of activity^37^. Using tag data, we calculated the work done to get insight into the energy expended during each part of the foraging dive.

Energy expended by the animals was calculated as the integral of power over time. We calculated power from swimming speed and minimum specific acceleration (the part of acceleration that is created through active propulsion by the animal), both estimated from the tag data, and the whales’ body mass. Because the actual mass of the whales was unknown, we averaged mass estimates from the literature^1,38^ and used these estimates to come to a relative energy expenditure that could be compared between individuals. Note that the actual body masses may have differed between the two animals, so the results presented here should be considered relative to weight.

**Figure 6:**
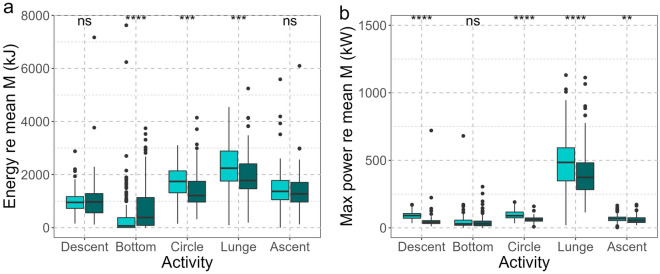
(**a**) Energy expended and (**b**) estimated maximum power usage relative to a mean body mass for each part of a foraging dive for Edna (turquoise) and Milky Way (dark green). Boxes show 25–75% quantiles, horizontal solid lines represent the median. Whiskers indicate 5–95% confidence intervals, with dots representing outliers outside of that range. Significant differences between animals are indicated as: *ns* not significant, *p < 0.05, **p < 0.01, ***p < 0.001, ****p < 0.0001.

Relative energy expenditure varied throughout the different parts of the dive and between the two whales. Lunges were the most energetically costly parts of a foraging dive, while the least amount of energy was expended while swimming at depth (Fig. 6a). Lunging was a high-power activity for both animals (Fig. 6b). Edna produced more power during lunges and expended more energy relative to body weight on a lunge than Milky Way. When summing energy use over an entire foraging dive, Edna also expended more energy relative to body weight than Milky Way (Fig. S2). For both animals, increased energy expenditure during a dive was correlated with higher breathing rates (Fig. 4; generalized linear model (GLM): energy expenditure estimate = 3.5 × 10^–7^, SE = 1.0 × 10^–7^, p < 0.001).

## Discussion

This study provides the first in-depth descriptions of the foraging behavior of two Rice’s whales in the Gulf of Mexico using kinematic tags. As a resident species of the Gulf of Mexico, Rice’s whales are dependent on local food sources, and may be impacted by the numerous anthropogenic activities taking place in this area. Both Rice’s whales conducted foraging dives predominantly during the day, and stayed close to the surface at night, putting them at risk of vessel strike. In contrast to the more variable lunging depth of Bryde’s whales, foraging consisted predominantly of lunging at depth, preceded by circling behavior in half to two-thirds of lunges. Both animals had low lunge rates (1–2 lunges per dive), which is lower than that of similar-sized, krill eating rorquals, and more commonly found for fish-eating rorquals (Table S1). In general, both animals expended most energy during lunging and circling, reaching swimming speeds similar to those of other small rorqual species^14,39^.

Although the overall foraging patterns of Edna and Milky Way were similar, a detailed analysis of the results showed that Edna was foraging more than Milky Way. Edna expended an equal amount of energy relative to body mass per foraging dive compared to Milky Way, but performed more foraging dives than Milky Way in a third of the time. Edna also had a higher breathing rate when surfacing. One possible cause is the association of Milky Way with a small animal, as female humpback whales associated with a calf lowered their energy expenditure compared to unassociated females^40^. On the other hand, the shallower point of neutral buoyancy for Edna suggests that Edna may have had smaller lipid stores than Milky Way at the time of tagging. Although the lack of body measurements made it impossible to come to reliable energy measurements, the higher breathing rate after foraging dives supports the increased energy use relative to body mass of Edna compared to Milky Way.

### Lunge behavior

The average lunge rate of one to two lunges per dive for both tagged Rice’s whales is lower than would be expected based on previous studies of rorqual foraging behavior^5,15,41,42^. Rorquals were found to lunge more when foraging > 100 m^19,20^, and smaller rorqual species typically perform more lunges per foraging dive than larger species, which is probably caused by positive allometry of the buccal pouch and the trade-off between the relative size of the buccal pouch and the energy cost of one lunge^9^. However, those studies focused on krill-eating rorquals, which have higher feeding rates due to the small maneuverability of their prey.

Based on the lunge behavior, Rice’s whales are likely foraging on fish. Feeding rates of 4–9 lunges per hour, as found in this study, are more consistent with the rates found for fish-eating rorquals (Table S1). Both humpback whales and Bryde’s whales, that feed on both fish and krill, had lower lunging rates when feeding on fish than on krill^15,28^. Feeding rates of fish-eating South African Bryde’s whales were similar to those of Rice’s whales in shallow waters, but went up in deeper waters, where the Bryde’s whales made 4–5 lunges per 10 min dive (P. Segre, pers. comm.). Rice’s whales also showed circling behavior, like the South African Bryde’s whales that chased fish along the seafloor, reaching speeds that were comparable to the maximum speeds reported here (5.3 m/s for Bryde’s whales^14^ vs 5.6–6.1 m/s, this study). An alternative explanation for the low foraging rates could be related to prey density. Prey densities could have been low or have exhibited spatial clustering, which would lead to a different cost–benefit analysis, favoring shorter dives with few lunges to minimize oxygen use^20^.

### Anthropogenic threats

Various aspects of Rice’s whale foraging behavior found here pose concern for the species’ survival. First, low lunge rates compared to what has been found for closely related species suggest that these animals might not have an optimal energy gain. Diving deep is energetically costly and is associated with a higher lunge rate than shallow diving in other rorquals^19^. Second, the high-power circling along the seafloor before lunging upward by a Rice’s whale might cause an increased risk for encountering and becoming entangled in bottom-longline fishery gear, which is the most common fishery in the Rice’s whale core habitat^6^. Bryde’s whales performing similar high-speed chases along the seafloor had an increased risk of entanglement in fishing gear^14^, which could also be the case for Rice’s whales. Third, the tendency of both whales to spend night time predominantly within 15 m of the surface supports earlier voiced concerns that they have a high risk of collision with vessels^6^. With two tagged animals, the sample size of this study is small, but the above-mentioned observations in their behavior, as well as the small population size, give serious cause for concern for the long-term survival of the species. The population was last estimated at ~ 51 individuals^3^, so any direct impact on their fitness could lead to a swift extinction.

## Conclusion

This study is the first detailed documentation of Rice’s whale foraging behavior. Although the sample size was small, similarities in foraging behavior between the two individuals make it possible to draw some preliminary conclusions about Rice’s whale foraging behavior. Both animals lunged less frequently than previously studied rorqual species, which could be related to their prey type and abundance, or could be related to differences in foraging habitat. Additionally, bottom-foraging with circling during the day and prolonged surface resting at night may put these animals at risk of entanglement and vessel collision. In combination with the poor body condition of both animals and the small population size, these results come as a warning signal. To enhance the survival probability of the species, more research to inform effective conservation management is warranted.

## Methods

### Ethics statement

Data were collected under NMFS permits 17312 issued to the Scripps Institution of Oceanography and under Marine Mammal Research Permits 14450-03 and 14450-05 issued to the Southeast Fisheries Science Center by the NOAA Fisheries Office of Protected Resources, Permits Division. All data collection was approved by an independent animal experiments committee.

### Field methods

Rice’s whales were tagged with a suction-cup-attached Acousonde tag (Greeneridge Sciences, Inc.) using the NOAA Ship *Gordon Gunter* to search for whales and the vessel’s 7-m Rigid Hull Inflatable Boat (RHIB) *R3* to approach the free-ranging animals. The Acousonde tag was attached to the whale using a hand-held pole deployment method. After tag attachment, the whales were tracked by observers on the *Gordon Gunter* when possible, during daylight hours. A VHF receiver on the tag allowed it to be detected when it was above the water surface and provided direction for recovery after the tag detached from the whale. Milky Way was tagged in 2015 between 20 September 09:25 and 23 September 01:10 (CDT) at 29.261° N and 86.268° W, and in 2018, Edna was tagged on 3 July 17:16 at 28.757° N and 85.705° W with the tag remaining attached until 4 July 18:25 (CDT). The water depth at the location of tagging was determined post hoc from online bathymetry data^43^.

The Acousonde (Model B003B) tag includes temperature and pressure sensors, triaxial magnetometers and accelerometers, and a hydrophone. The data sampling differed between the two tag deployments. In 2015, all non-acoustic sensors were sampled at 5 Hz. In 2018, the sampling rates were: temperature at 5 Hz, pressure at 10 Hz, accelerometer at 800 Hz, and magnetometer at 40 Hz. Acoustic data were sampled at 9110 Hz during both deployments. For consistency in analyses, the accelerometer and magnetometer data from 2018 were down-sampled to 10 Hz.

### Tag data analysis

The accelerometer, magnetometer, and pressure data were calibrated and corrected for changes in tag placement using the *tagtools* package (animaltags.org) in *Matlab* (MATLAB 2016b, Mathworks, Natick, MA). Pitch and roll were calculated from the accelerometer data, and heading was calculated from the combination of the accelerometer and magnetometer data. The tag depth sensor was temperature corrected using periods when the whale was at the surface before and after deep dives when the tag was temperature equilibrating. The correction was performed using the *fix_pressure* function from the *tagtools* package. Dives were detected automatically using the *find_dives* function from the *tagtools* package, with a minimum dive depth set at 10 m.

Tag data were manually analyzed using the Matlab-based *Triton* software package^44^ (version 1.0 2021 09 21, https://github.com/MarineBioAcousticsRC/Triton) with a customized add-on “remora” software module, *MTViewer* (inspired by Burgess MT Viewer, www.acousounde.com), that synchronizes displays of data from acoustic and kinematic (pressure, orientation) sensors and includes a tool for annotating events. The following events were manually annotated and occurrence times were extracted for further analysis: breaths, depth of neutral buoyancy, circling, foraging lunge, descent end, and ascent start. Breaths were identified by both minima in depth and the broadband sound of exhalation/inhalation on the hydrophone. We subdivided breaths in inhalation and exhalation by the break in the recording due to the tag exiting the water, and only inhalation times were used in further analysis.

We used a proxy for estimating neutral buoyancy by identifying the depth during both descent and ascent at which fluking stopped and gliding downward or upward (respectively) began. Neutral buoyancy was then calculated as the average of those points. Circling was identified by a continuous ≥ 180° change in heading, while pitch was < |85|° to avoid periods of gimbal lock. Foraging lunges were identified as the point at which a sharp increase in speed occurred, followed by a rapid deceleration. Lunges typically also contained large changes in pitch and roll, which helped to identify them. Descent end and ascent start were the points between which the dive leveled to a relatively narrow depth range, near the maximum depth of the dive. Swimming at depth that was not annotated as either circling or lunging, was assigned to “bottom”. Following annotation, we extracted timing information for each event for further analyses using custom Matlab-based routines. Events were assigned to day or night periods based on the time of "civil" twilight, when the sun was at 6° below the horizon (http://users.softlab.ntua.gr).

### Estimation of energy expenditure

To get an estimate of the minimum energy required by Rice’s whale to sustain their foraging behavior, we calculated the energy expended as work done during foraging using the minimum specific acceleration of the animals. Work done was calculated as:

1 $$work \, done= {\int }_{0}^{t}P$$

where P = power, estimated as:

2 $$P=Mav$$

and M = body mass, a = minimum specific acceleration and v = velocity. We estimated mass as an average body mass of 6000 kg, from a generally accepted length-to-weight conversion for baleen whales, using species-specific constants for the closely related Bryde’s whales (a = 0.012965, b = 2.74)^38^. Length was calculated as the average length (9.2 m) from documented Rice’s whales^1^.

Acceleration was calculated as the minimum specific acceleration. Measured acceleration consists of acceleration due to propulsion (known as the specific acceleration), body rotation and gravity. The minimum acceleration that is due to specific acceleration, called the minimum specific acceleration^45^ is calculated by combining the data streams from the magnetometer and accelerometer. The magnetometer only measures body rotation, which makes it possible to separate the acceleration component that is due to body rotation from the component that is due to propulsion. We calculated the minimum specific acceleration sensu Martín Lopéz et al.^45^.

We estimated velocity as the whale’s swimming speed from the tag jiggle (high-frequency vibrations of the tag on the animal that increase with speed), which was regressed against the tags’ accelerometers’ Orientation-Corrected Depth Rate (OCDR) for verification, following Cade et al.^46^. Because the speed estimation was most reliable with a sampling rate of > 5 Hz, speed was calculated for 2018 first, against which the speed values of 2015 were checked for reliability. The speed values of the 2015 tag were similar to those of the 2018 tag and had an R^2^ of 0.5–0.61 with OCDR, so they were considered reliable (Fig. S4).

### Statistics

To compare dive characteristics between individuals, we tested whether the measured variables were normally distributed using a Shapiro–Wilk test of normality. As none of the variables were normally distributed, we used a Wilcoxon rank test to compare measurements between individuals. We investigated the effect of activity (descent, circle, lunge, ascent) and individual on power and energy expenditure with linear models. For power, we used a Gaussian-distributed model with log transformation of power to correct for non-linearity in the data, while for energy expenditure, we used a generalized linear model (GLM) with a Gamma distribution. We investigated the influence of energy expenditure and individual on breathing rate using a GLM with quasipoisson distribution. Additionally, we tested for the differences in dive depth of lunges preceded by a circle vs lunges that were not preceded by a circle using a linear model. All models were checked against violations of the model assumptions with model diagnostics, and final models were obtained through dredging (*MuMIn* pacakage), an automated way of selecting the most parsimonious model that corrects for order in variable selection. All statistical tests were performed in R 4.1.2 (R Core Team 2022).

## Supplementary Information

Supplementary Information.

## Data Availability

The data is available in Dryad: https://datadryad.org/stash/share/60qJhCm2w0pEY9JahhPtVURXn0SgNoZ9PdjDQHAjG1Y.
