# Supplementary material for: Kinematics and energetics of foraging behavior in Rice’s whales of the Gulf of Mexico
Source: Sci Rep. 2023 Jun 2;13:8996. doi: 10.1038/s41598-023-35049-z (PMC10238412; doi:10.1038/s41598-023-35049-z)
Supplement: Supplementary file 1 — Supplementary Information. [file 41598_2023_35049_MOESM1_ESM.pdf]

Supplementary Information

Kinematics and Energetics of Foraging Behavior in Rice's Whales of the Gulf of Mexico

Annebelle CM Kok[1*], Maya J. Hildebrand[1], Maria McArdle[1], Anthony Martinez[2], Lance P. Garrison[2], Melissa S. Soldevilla[2] and John A. Hildebrand[1]

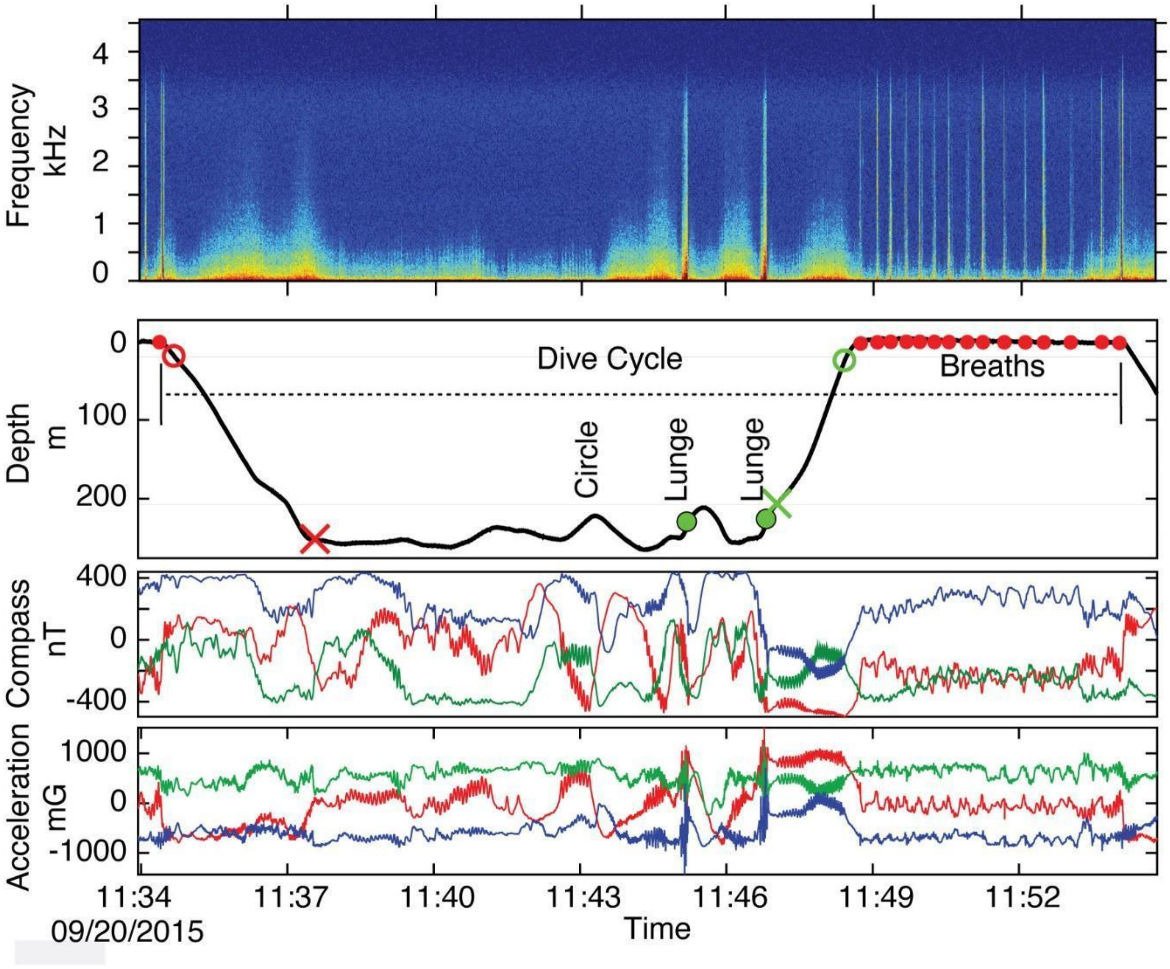

Supplementary Figure S1. Acoustic spectrogram, depth, compass, and acceleration records for one dive cycle of Milky Way with annotations for breaths (red dots), neutral buoyancy (red and green circles), foraging lunges (green dots), end of descent (red cross), and beginning of ascent (green cross).

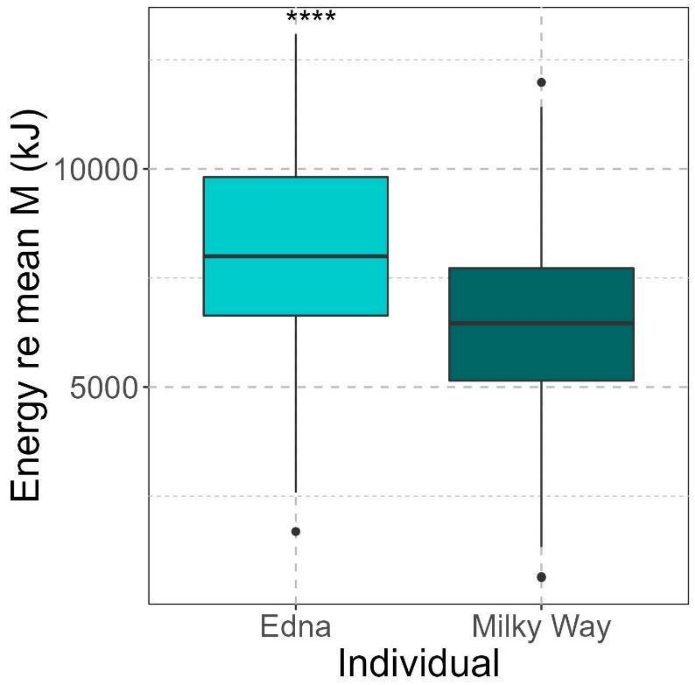

*Supplementary Figure S2. Energy usage relative to mean body weight per foraging dive for Edna (turquoise) and Milky Way (dark green). Energy expenditure was significantly higher for Edna (Kruskal-Wallis, χ^2 = 18.332, p<0.001) . Significant differences between animals are indicated as: *** = p<0.001.*

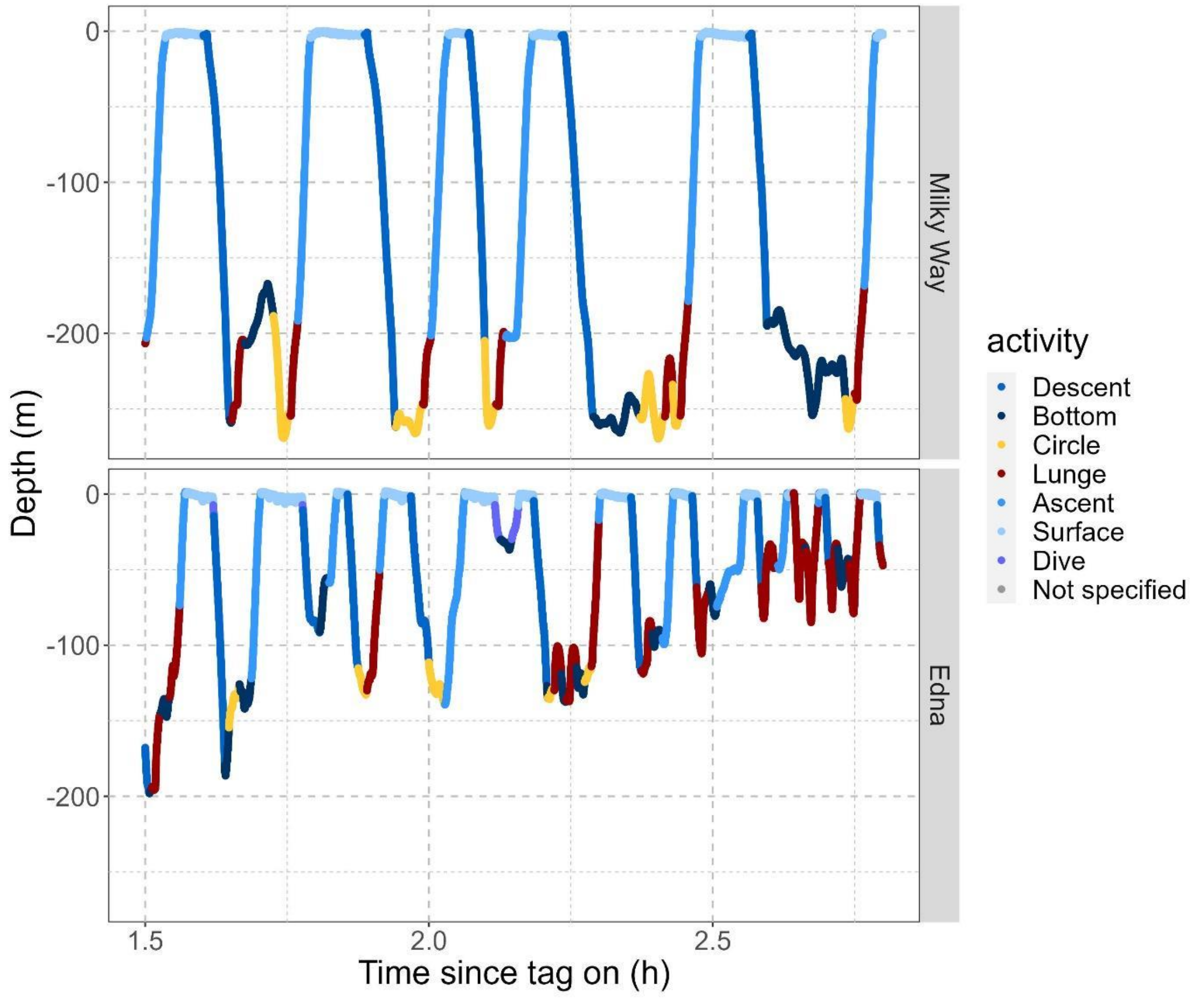

Supplementary Figure S3. Section of the dive profile of both tagged animals, color-coded by assigned activity. The type of activity was based on depth, swimming speed, and pitch, roll and heading of the animal.

a)

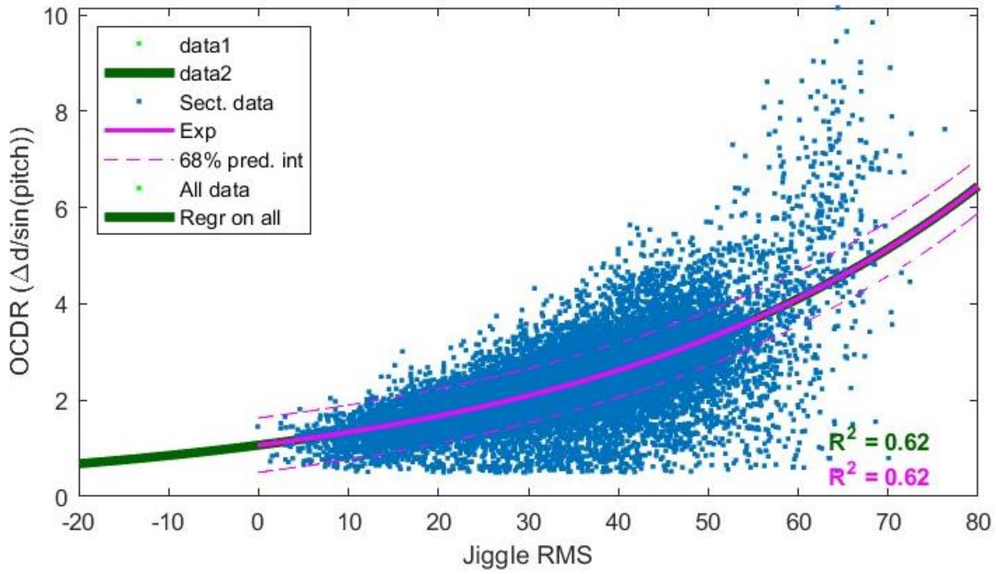

b)

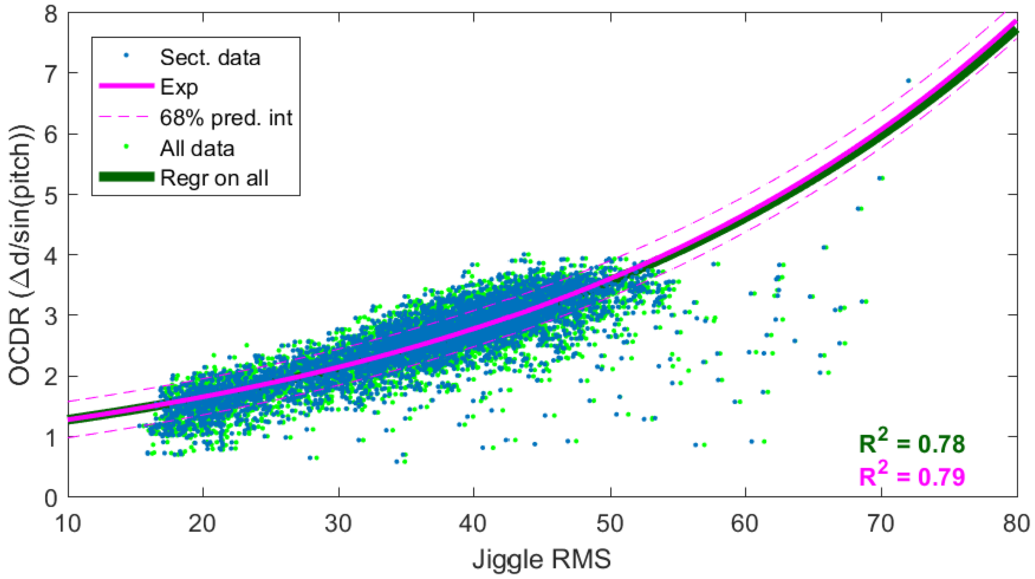

Supplementary Figure S4. Regression of the tag jiggle root-mean-square against the orientation corrected depth rate (OCDR) for a) Milky Way (G2015) and b) Edna (G2018). Green dots = all data points, blue dots = data points of one section of the tag, pink solid line = expected correlation between Jiggle RMS and OCDR, pink dashed line = 65% of predicted values fall within these lines, green solid line = regression based on data. Sensu Cade et al. 2018.

Supplementary Table 1:

Reference	Species	Prey type	Lunges/dive	Lunges/10 min	Lunges/hour mean (sd)
(Izadi et al., 2022)	Bryde's whale	krill	5.5		11.2 (9)
		fish	low		
(Segre et al., 2022)	Bryde's whale	fish	1 (shallow)<br>4-5 (deep)	4-5 (deep)	
(Alves et al., 2010)	Bryde's whale		1-3		
(Owen et al., 2017)	Humpback whale	krill		10	49
		fish		3	5
(Burrows et al., 2016)	Humpback whale	krill	8-10		
(Cade et al., 2020)	Humpback whale	fish			3.9 (2)
(Friedlaender et al., 2020)	Fin whale	krill	4		15.15 (4.9, shallow)<br>29.33 (1.5, deep)

Alves, F., Dinis, A., Cascão, I., & Freitas, L. (2010). Bryde's whale (Balaenoptera brydei) stable associations and dive profiles: New insights into foraging behavior. *Marine Mammal Science*, *26*(1), 202–212. https://doi.org/10.1111/j.1748-7692.2009.00333.x

Burrows, J. A., Johnston, D. W., Straley, J. M., Chenoweth, E. M., Ware, C., Curtice, C., de Ruiter, S. L., & Friedlaender, A. S. (2016). Prey density and depth affect the fine-scale foraging behavior of humpback whales Megaptera novaeangliae in Sitka Sound, Alaska, USA. *Marine Ecology Progress Series*, *561*, 245–260. https://doi.org/10.3354/meps11906

Cade, D. E., Carey, N., Domenici, P., Potvin, J., & Goldbogen, J. A. (2020). Predator-informed looming stimulus experiments reveal how large filter feeding whales capture

highly maneuverable forage fish. *Proceedings of the National Academy of Sciences of the United States of America*, *117*(1), 472–478. https://doi.org/10.1073/pnas.1911099116

Friedlaender, A. S., Bowers, M. T., Cade, D., Hazen, E. L., Stimpert, A. K., Allen, A. N., Calambokidis, J., Fahlbusch, J., Segre, P., Visser, F., Southall, B. L., & Goldbogen, J. A. (2020). The advantages of diving deep: Fin whales quadruple their energy intake when targeting deep krill patches. *Functional Ecology*, *34*(2), 497–506. https://doi.org/10.1111/1365-2435.13471

Izadi, S., Aguilar de Soto, N., Constantine, R., & Johnson, M. (2022). Feeding tactics of resident Bryde's whales in New Zealand. *Marine Mammal Science*, 1–14. https://doi.org/10.1111/mms.12918

Owen, K., Kavanagh, A. S., Warren, J. D., Noad, M. J., Donnelly, D., Goldizen, A. W., & Dunlop, R. A. (2017). Potential energy gain by whales outside of the Antarctic: prey preferences and consumption rates of migrating humpback whales (Megaptera novaeangliae). *Polar Biology*, *40*(2), 277–289. https://doi.org/10.1007/s00300-016-1951-9

Segre, P. S., di Clemente, J., Kahane-Rapport, S. R., Gough, W. T., Meÿer, M. A., Lombard, A. T., Goldbogen, J. A., & Penry, G. S. (2022). High-speed chases along the seafloor put Bryde's whales at risk of entanglement. *Conservation Science and Practice*, *e12646*, 9. https://doi.org/10.1111/csp2.12646
